# Serum Cystatin C as a Potential Marker for Glomerular Filtration Rate in Patients with Cholangiocarcinoma

**DOI:** 10.18502/ijhoscr.v14i3.3723

**Published:** 2020-07-01

**Authors:** Mang Ngaih Ciin, Tanakorn Proungvitaya, Temduang Limpaiboon, Sittiruk Roytrakul, Ubon Cha’on, Doungdean Tummanatsakun, Siriporn Proungvitaya

**Affiliations:** 1 Centre of Research and Development of Medical Diagnostic Laboratories (CMDL), Faculty of Associated Medical Sciences, Khon Kaen University, Khon Kaen 40002, Thailand; 2 Cholangiocarcinoma Research Institute (CARI), Faculty of Medicine, Khon Kaen University, Khon Kaen 40002, Thailand; 3 National Center for Genetic Engineering and Biotechnology, National Science and Technology Development Agency, Pathumthani 12120, Thailand; 4 Department of Biochemistry, Faculty of Medicine, Khon Kaen University, Khon Kaen 40002, Thailand

**Keywords:** Cystatin C, Chronic kidney disease epidemiology collaboration (CKD-EPI) equation, Glomerular filtration rate, Cholangiocarcinoma

## Abstract

**Background:** Cholangiocarcinoma (CCA) is the second most common primary hepatobiliary cancer. These patients have meager prognosis and short-term survival. Precise assessment of glomerular filtration rate is a fundamental aspect of clinical care in cancer patients. Cystatin C has been proposed to be superior to creatinine, a well-known marker of renal function. This study aimed to evaluate cystatin C as a marker of GFR calculation in CCA patients.

**Materials and Methods:** One hundred thirty serum samples from CCA patients and 32 from controls were included in this study. Serum cystatin C was measured using immunoturbidity assay. Estimated glomerular filtration rate was calculated by three equations established by chronic kidney disease epidemiology collaboration (based on creatinine and/or cystatin C).

**Results:** Serum cystatin C in CCA patients was higher than that of controls (p=0.0002). Cystatin C was positively correlated with BUN in CCA group (p=0.019). eGFR based on cystatin C and based on both cystatin C and creatinine in CCA was low with significantly different from those of control (p<0.001). Although there was no difference in eGFR using three equations in control, creatinine based eGFR was high with significantly different from eGFR based on cystatin C and on both creatinine and cystatin C in CCA (P=0.000). Proportion in each eGFR stage by three equations showed a high sensitivity with significantly different in CCA (p<0.05).

**Conclusion:** There was a high sensitivity of cys C with significant difference between creatinine and/or cystatin C based eGFR in CCA patients. It should be taken into consideration of mild changes in eGFR by cystatin C which is important in managing drug dosage for CCA patients.

## Introduction

 Cholangiocarcinoma (CCA) is the second most common primary hepatobiliary cancer originating from epithelial cells lining the intra- and extrahepatic biliary tracts. It is more common in Southeast Asia, especially in the Greater Mekong Subregion (GMS), than in Western countries. The most important risk factor for CCA in GMS is the infection with *Opisthorchis viverrini* (OV) and *Clonorchis sinensis* which are ingested by eating raw or undercooked fish^1^^, ^^2^. Patients with CCA have a meager prognosis and short survival time. Most patients have unresectable diseases at the time of diagnosis and usually die within 6-12 months from the cancer cachexia, liver failure and biliary sepsis^3^. 

Renal impairment is a relatively common complication in cancer patients with therapeutic drugs, irrespective of the type of malignancy^4^. For this reason, monitoring renal function of cancer patients is essential for the safe administration and follow-up of therapeutic agents. The glomerular filtration rate (GFR) is regarded as the superlative parameter for estimating kidney function^5^ and an accurate GFR assessment is essential for the better clinical care for cancer patients. GFR underestimation might cause misidentification of some patients unsuitable for chemotherapy such as cisplatin therapy, or expose them to underdosing, influencing survival outcomes^6^^,^^7^. 

Serum creatinine has long been used for eGFR calculation because it is an easily measurable and widely available marker for renal function. However, serum creatinine level is influenced by various factors such as muscle mass, body weight, and gender, which can affect the utility of serum creatinine level in practice^8^. In bladder cancer patients, 40% of those having decreased GFR determined by inulin clearance showed normal serum creatinine level^9^. It remains unclear whether eGFR based on serum creatinine can accurately forecast GFR in cancer patients because cancer patients often suffer complications from emaciation and muscle loss associated with cachexia, anorexia and mal-function of the gastrointestinal tract that may cause noteworthy changes in creatinine production^10^. 

As an alternative of serum creatinine level, recently cystatin C (cys C) has been highlighted as a new marker for estimation of GFR ^11^^,^^12^. Cys C is a nonglycosylated low molecular weight protein (13 kDa) and is produced at a constant rate by all nucleated cells. It is freely filtered by the glomerulus and completely reabsorbed by the proximal tubular cells^13^. Cys C has been proposed to be potentially superior to serum creatinine for estimating renal function^14^. Recent studies in gastric cancer, and head and neck cancer suggest that serum cys C may serve as a reliable alternative clinical marker of renal function^15^^, ^^16^. However, in some cancer patients, the serum cys C is considered not appropriate for assessing renal function because cancer cells themselves produce cys C^17^ and increase in serum cys C levels can affect the eGFR level resulting in underestimation of true GFR^18^. The present study is, therefore, aimed to evaluate cys C as a marker of GFR calculation in CCA patients for appropriate treatment. 

## MATERIALS AND METHODS

**Sample collection**

A total of 130 CCA serum samples with clinical data (age, sex, creatinine, survival days, blood urea nitrogen (BUN), cholesterol, total and differential protein, liver function tests (LFT)) were obtained from the Cholangiocarcinoma Research Institute (CARI), Faculty of Medicine, Khon Kaen University, Thailand. As controls, 32 serum samples were obtained from the persons who went to check-up at the Office for Medical Technology and Physical Therapy Health Service, Faculty of Associated Medical Sciences, Khon Kaen University. The serum samples were kept at 20˚C until further use. This project was approved by the Khon Kaen University Ethics Committee for Human Research (HE611409).

**Cystatin C measurement**

Serum cys C level was measured using an immunoturbidity assay kit (Diazyme, California, USA). Cys C in the sample binds to latex particles coated with the specific anti-cystatin C antibody to cause agglutination. The degree of the turbidity caused by agglutination can be measured optically and is proportional to the amount of cys C in the sample. In this assay, 3 µl of serum was used and the absorbance was read at 540 nm wavelength. This was an end point assay, where 5 calibrators and 2 levels of controls (High and Low internal controls) provided by the manufacturer were included.

**eGFR calculation**

eGFR was calculated using three Chronic Kidney Disease Epidemiology Collaboration (CKD-EPI) equations based on the creatinine, cys C and both creatinine and cys C^19^. 

**Statistical analyses**

Statistical analysis was performed using IBM SPSS 22 Statistics (The International Business Machines Corporation, Charles Ranlett Flint, Armonk, New York, U.S.) and GraphPad Prism v.5 software (GraphPad Software Inc., La Jolla, CA, U.S.). The difference between two groups was estimated using the Mann-Whitney tests. Linear regression test was used to assess the correlation of cystatin C with other renal markers. Kruskal Wallis test was used to assess the comparison between creatinine and/or cys C in assessing GFR classification based on their stages. Blant-Altman analysis was used to determine the agreement between cystatin C and creatinine in assessing the eGFR.* P* value <0.05 was considered statistically significant.

## Results

**Clinical characteristics of CCA patients and controls**

**Figure 1:**
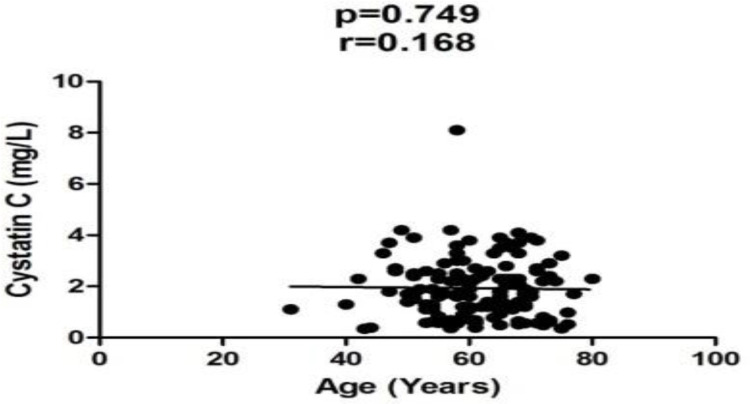
Relationship between age and serum level of cystatin C in CCA group by Pearson’s correlation. p<0.05 as significance.

Clinical characteristics of the participants are shown in Table 1. Serum cys C level of CCA group (1.93±1.2 mg/L) was significantly (p=0.0002) higher than that of control group (1.1±0.59 mg/L). The mean ALP, ALT and AST were higher in CCA than that of control group (p<0.05). CCA patients were older than control group (p<0.0001) but there was no correlation of serum cys C and age (p=0.749) (Figure 1). 

**Table 1:** Clinical characteristics of healthy control and cholangiocarcinoma groups

**Parameters**	**Control group** **(n=32)**	**CCA group ** **(n=130)**	**p-value**
Age	44.3±18.7 (19-85)	61.1±8.6 (31-80)	<0.0001*
**Renal function ** **tests**			
BUN (5.8-19.1 mg/dl) Creatinine (0.5-1.5 mg/dl) Cystatin C (0.5-1.03 mg/L)	13±2 (7-18) 0.8±0.15 (0.4-1.4) 1.1±0.59 (0.2-3.1)	12.5±3.7 (5.3-39) 0.85±0.15 (0.4-3.8) 1.93±1.2 (0.35-8.1)	0.743 0.981 0.0002*
**Liver function ** **tests**			
ALT (4-36 U/L) AST (12-32 U/L) ALP (42-121 U/L)	21.6±9.0 (8-43) 27±6.5 (19-85) 51±7 (37-87)	37±21.5 (9-611) 42±16.3 (14-1077) 166.5±84.3 (24-1005)	<0.0001* 0.0002* <0.0001*

a: Value represents median** ± **Quartile deviation and (min-max).

b: Value represents mean** ± **SD and (min-max). cysC, Cystatin C; BUN, Blood urea nitrogen; ALP, Alkaline phosphatase; ALT, Alanine aminotransferase; AST, Aspartate aminotransferase. The different values among two independent sample groups were estimated using Mann-Whitney test.

* Significant difference between control and CCA.

**Correlation of cystatin C with BUN and creatinine**

**Figure 2:**
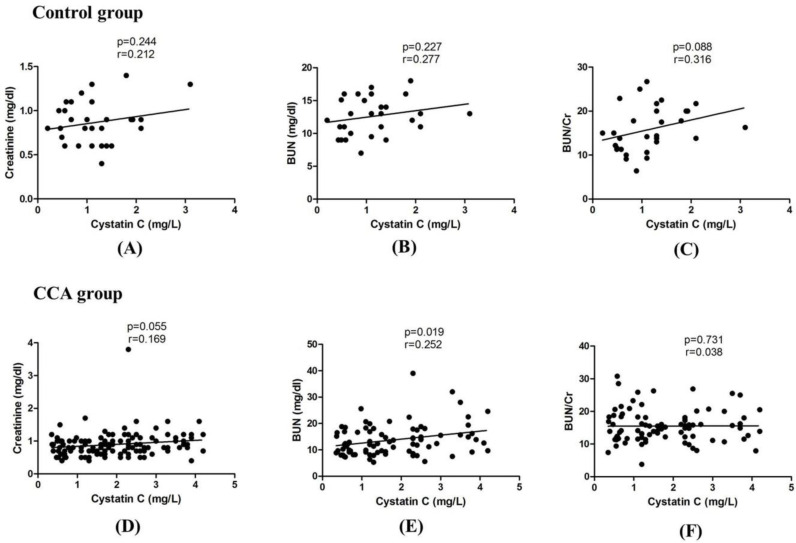
Correlation between serum cys C and creatinine, cys C and BUN, and cys C and BUN/Cr by linear regression test. (A), (B) and (C) represent control group and (D), (E) and (F) were CCA group. p<0.05 considered as statistically significance.

In control group, there was no correlation of serum cys C with creatinine (Cr) and blood urea nitrogen (BUN) which are the well-known markers for renal function. In contrast, serum cys C level in CCA group was significantly correlated with BUN (p=0.019) but not with Cr. Blood urea nitrogen to Cr ratio (BUN/Cr) has been reported as an independent factor reflecting dietary protein intake at each level of renal function. Therefore, the correlation of serum cys C with BUN/Cr was analyzed in this study and no correlation were observed in both control and CCA groups (Figure 2). 

**eGFR using three CKD-EPI equations**

**Figure 3:**
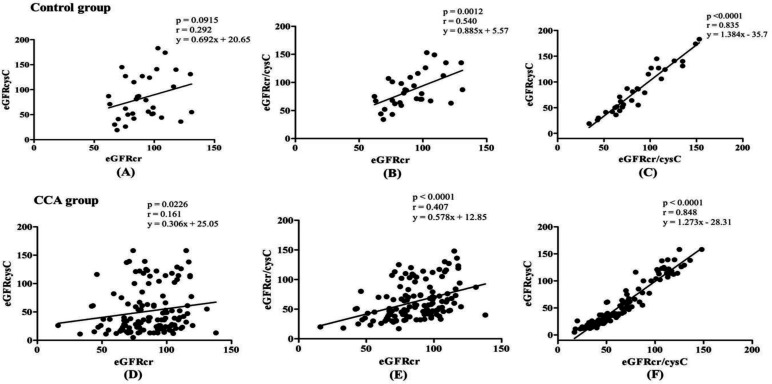
The regression line by Spearman correlation analysis among eGFRs using three equations. (A), (B) and (C) represent control group and (D), (E) and (F) were CCA group. p<0.05 considered as statistically significance. eGFRcr: eGFR based on creatinine, eGFRcysC: eGFR based on cys C and eGFRcr/cysC: eGFR based on both creatinine and cys C.

The mean eGFR based on serum cys C and combined creatinine and cys C in CCA were high sensitivity with significantly different from those of control group (p<0.001) (Table 2). The correlation analysis among each equation was done in both control and CCA group by Spearman’s correlation. There was positive correlation among each equation in CCA group (p<0.05) but creatinine based eGFR did not correlate with cys C based eGFR in control group (p=0.092) (Figure 3).

**Table 2:** Comparing eGFR by three equations between control and CCA groups

**Parameters**	**Control group** **(n=32)**	**CCA group** **(n=130)**	**p-value**
eGFRcr (≥90 ml/min/1.73m^2^)	91.3±18.9^b^ (62-131)	86.2±21.1^b^ (16-138)	0.382
eGFRcysC (≥90 ml/min/1.73m^2^)	83.9±44.7^b^ (19-183)	36±21.5^a^ (5-158)	<0.001*
eGFRcr/cysC (≥90 ml/min/1.73m^2^)	86.4±30.9^b^ (34-153)	53±17.5^a^ (17-148)	<0.001*

a : Value represents median** ± **Quartile deviation and (min-max).

b : Value represents mean** ± **SD and (min-max). eGFRcysC: eGFR based on cys C, eGFRcr: eGFR based on serum creatinine, eGFRcr/cysC based on both creatinine and cys C. Man-Whitney test.

* Significant difference between control and CCA.

**Agreement among eGFRs by equations based on creatinine and/or cys C**

**Figure 4:**
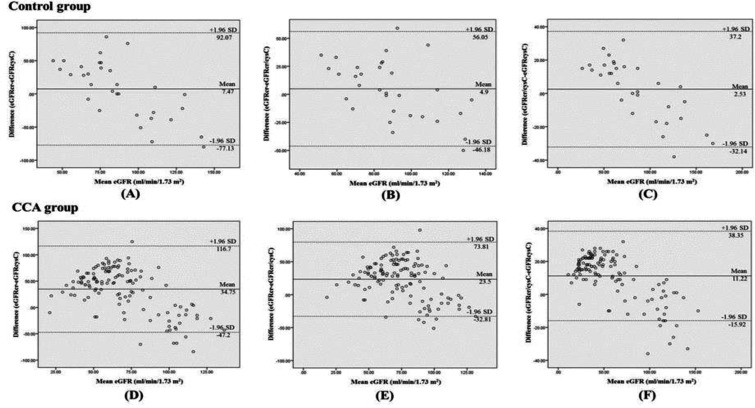
Agreement between eGFR based on Cys C and/or Creatinine using Blant-Altman analysis. (A), (B) and (C) represent control group (mean difference, p>0.05) and (D), (E) and (F) were CCA group (mean difference, p<0.0001). p<0.05 as significance.

The agreement between the eGFR based on creatinine and/or cys C was studied using Blant-Altman analysis. eGFRs using creatinine and/or cys C was not significantly different in control group (p>0.05). However, eGFR based on creatinine and/or cys C was high sensitivity with significantly different in CCA group (P<0.0001) (Figure 4).

**Classification of eGFR by three equations**

Table 3 indicated the difference in eGFR stages using CKD EPI equations based on creatinine and cystatin C. Based on KDIGO guidelines, we categorized patients into five groups for each of the three equations: eGFR ≥ 90 mL/min/1.73m^2^, eGFR = 60-89 mL/min/1.73m^2^, eGFR = 30-59 mL/min/1.73m^2^, eGFR = 15-29 mL/min/1.73m^2^ and eGFR < 15 mL/min/1.73m^2^ (Stage 1,2,3,4 &5). 

According to the National Kidney Foundation-Kidney Disease: Improving Global Outcomes (NKF-KDIGO) guidelines, the normal GFR range is ≥90 ml/min/1.73m^2^ in adults^20^. In control group, normal eGFR level (≥90 ml/min/1.73m^2^) was estimated in 46.9% by creatinine based equation and in 35% by equations based on cys C and both creatinine and cys C. The proportion difference among three equations was significant (p=0.003). 

Almost 50% of CCA patients showed normal eGFR level by creatinine-based equation while only 20% of CCA patients were estimated for normal eGFR level using the equations based on cys C and both creatinine and cys C (p<0.0001). Although 43.1% was classified as CCA patients with severe decreased eGFR level (stage 4 and 5) by cys C based equation, the proportion by creatinine and both creatinine and cys C based equations was less than 10%. Moreover, the proportion in each stage by three equations was significantly different (p<0.05). 

**Table 3:** Difference in the stages of eGFR between creatinine and/or cys C

**Equations**	**eGFR (ml/min/1.73m** ^2^ **)**				
	**Stage 1 ** **(≥90)**	**Stage 2 ** **(60-89)**	**Stage 3 ** **(30-59)**	**Stage 4 ** **(15-29)**	**Stage 5 (<15)**
Control (n=32)					
CKD-EPIcr CKD-EPIcysC CKD-EPIcr/cysC	15 (46.9%) 11 (34.3%) 12 (37.5%)	17 (53.1%) 8 (25%) 14 (43.7%)	- 10 (31.3%) 6 (18.8%)	- 3 (9.4%) -	- - -
p	0.003	0.607	0.744		
CCA (n=130)					
CKD-EPIcr CKD-EPIcysC CKD-EPIcr/cysC	62 (47.7%) 26 (20%) 25 (19.2%)	55 (42.3%) 15 (11.5%) 32 (24.6%)	12 (9.2%) 33 (25.4%) 66 (50.8%)	1 (0.8%) 42 (32.3%) 7 (5.4%)	- 14 (10.8%) -
p	<0.0001	0.0008	<0.0001	0.0013	

The different values among three sample groups were estimated using Kruskal Wallis test.

## Discussion

 In the present study, we compared eGFRs calculated using three CKD-EPI equations in CCA and control groups. Serum creatinine and eGFR based on creatinine in CCA patients did not differ from those of control group in our study. In contrast, there were significant difference in mean eGFR based on cys C and both on creatinine and cys C since serum cys C level was significantly higher in CCA (1.93±1.2 mg/L) than that of control group (1.1±0.59 mg/L). Some studies reports that the serum level of cys C can increase earlier than some other materials such as serum creatinine and therefore may be more valuable than serum creatinine for early detection of renal dysfunction^21^^, ^^22^. A large scale meta-analysis conducted on report in 2002 demonstrated that serum cys C was superior to serum creatinine as a marker of GFR^14^.

**Figure 5:**
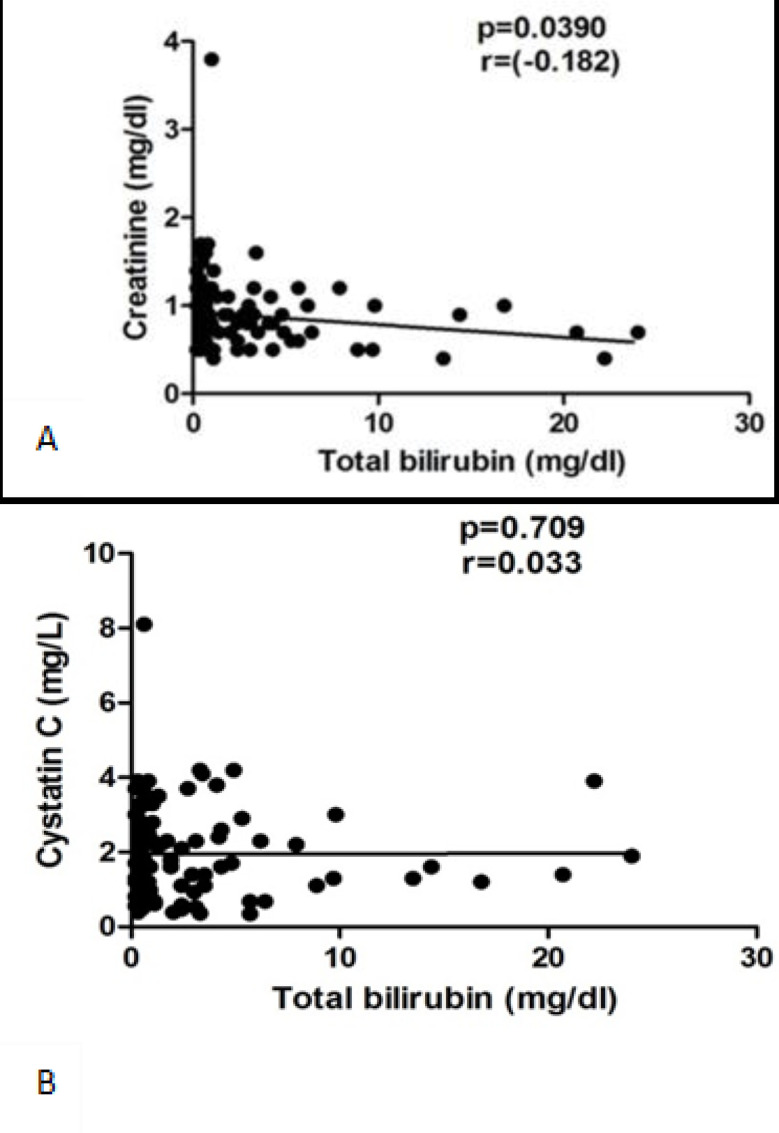
Correlation between serum creatinine (using enzymatic method) (A) and serum cystatin C (B) levels with serum total bilirubin levels

There was no correlation between serum creatinine and cys C in both CCA and control groups in our study. Although cys C level was higher than normal range in both groups, serum creatinine has not been noticeably increased. Serum creatinine levels are affected by both non-renal physiological factors such as muscle mass, body weight, gender which can affect its utility in practice ^9^. Moreover, measurements of serum creatinine levels can be affected by the amount of total bilirubin co-existing in the serum^23^^,^^24^. In fact, when we have examined the correlation between serum creatinine or serum cystatin C levels with serum total bilirubin levels (Figure 5), negative correlation was observed between creatinine and bilirubin, but not between cystatin C and bilirubin levels. In cancer patients, especially of hepatobiliary malignancies, therefore, serum creatinine should not be a “standalone” marker of renal function because these patients often suffer complications from emaciation and muscle loss associated with cachexia, anorexia and mal-function of the gastrointestinal tract and hyperbillirubinaemia that may cause noteworthy changes in creatinine production or creatinine measurements.

In this study, cys C showed a positive correlation with BUN in CCA group. Serum cys C is the product of a constitutive housekeeping gene, its synthesis occurring at a steady state, the serum levels mainly affected by renal function^13^. BUN has been known for a marker of renal function and elevations in urea level occur with high protein intake. The variation of BUN production rate was not or less influenced by muscle mass, body weight and age that are the commonly complications occurred in cancer patients. The correlation between cys C and BUN from our study proved that cys C was a marker of renal function that cannot be influenced by muscle mass, age and body weight. 

Our study showed a positive correlation among each eGFR by three equations in CCA group. However, in control group, eGFR based on creatinine and cys C alone did not correlate with each other. Moreover, mean eGFR difference among each equation was significant in CCA but not in control group. Furthermore, almost half of the population (50%) was estimated as normal eGFR level by CKD-EPI equation based on creatinine in both CCA and control group while cys C based equation and both combination of creatinine and cys C based equations estimated normal eGFR level in less than 40% in control and 20% in CCA group. Furthermore, 43.1% of CCA patients were classified as severe stage of declined eGFR by cys C based equations while the proportion of severe stage of eGFR was less than 10% by other two equations. Our results suggested that creatinine could overestimate eGFR in CCA patients and cys C could estimate early changes of eGFR. Creatinine is considered as not a sensitive marker to detect mild and medium decrease of renal function, also known as ‘creatinine blind area’ so that the over estimation of GFR usually occurs and make lots of under diagnosed cases^25^. A study by Daniel Giglio in 2014 showed that CKD-EPIscr equation was precise in cancer patients with GFR ≥60 ml/min/1.73 m^2 ^^26^. 

Cys C is a reliable marker for monitoring kidney function in patients with normal kidney function receiving cisplatin-based chemotherapy^27^. New equations such as CKD-EPI equations based on cys C and/or serum creatinine have been proposed for clinical applications^28^. Several studies have shown that cys C is a more sensitive marker of decreased GFR than serum creatinine^29^^-^^31^. In addition, serum cys C was reported to have valuable potential for the monitoring of GFR in gastric cancer, and head and neck cancer^32^^,^^33^. However, in some cancer patients, the serum cystatin C is not appropriate for estimating GFR because of its production by cancer cells. Some studies have shown that cystatin C is not trustworthy for GFR marker in ovarian cancer because of its nature as a cysteine protease inhibitor^34^, and the concentration variations in serum cystatin C might underestimate eGFR in esophageal cancer^35^. Therefore, the role of cys C in CCA should be further investigated. 

Renal impairment is a relatively common complication occurring in cancer patients. Since 1970s, renal failure has been increasingly assessed in patients with cancer^36^. A study in 2004 revealed that one-third of cancer patients presented renal insufficiency^37^. There is little information to help clinicians using the most appropriate equation for calculating eGFR in cancer patients although accurate estimation of GFR is important to determine the dosages of chemotherapeutic agents. A few previous studies have mainly focused on genitourinary or gynecological cancers, and the data were exclusively from Western patients^38^^-^^40^. This study is the first to estimate GFR in CCA patients in GMS.

Our study has some limitations. The gold standard for GFR measurements such as inulin, 51Cr-EDTA, or iohexol clearance was lacked in this study. However, cys C was compared with the bronze standard, creatinine. Moreover, cys C and creatinine based eGFRs were calculated by CKD-EPI equations recommended by KIDGO guidelines. It is not clear whether this finding would still be applicable for the patients under chemotherapy treatment because the information of treatment and type of drugs has not been provided. However, a study in 2015 reported that cys C is still superior to creatinine in detection of early stages of renal dysfunction in cancer patients under treatment with cisplatin^41^.

In conclusion, there is a high sensitivity of cys C with significant difference between creatinine and cys C based GFR in CCA patients. The proportion difference was also observed in classifying eGFR stages by three equations in CCA patients. This can probably become the consideration to do the examination on cys C in CCA patients to receive appropriate chemotherapeutic treatment. 

## References

[1] Plentz RR, Malek NP (2015). Clinical presentation, risk factors and staging systems of cholangiocarcinoma. Best Pract Res Clin Gastroenterol.

[2] Sripa B, Kaewkes S, Sithithaworn P (2007). Liver Fluke Induces Cholangiocarcinoma. PLoS Med.

[3] Woradet S, Songserm N, Promthet S (2016). Health-related quality of life and survival of cholangiocarcinoma patients in northeastern region of Thailand. PLoS One.

[4] Cosmai L, Porta C, Gallieni M, Perazella M (ed) (2016). CKD as a Complication of Cancer. American Society of Nephrology. Journal of the American Society of Nephrology.

[5] Stevens LA, Lerma EV, Nissenson AR (2012). Measurement of glomerular filtration rate. Nephrology Secrets.

[6] Robinson R, Tait CD, Somov P (2016). Estimated glomerular filtration rate is unreliable in detecting renal function loss during follow-up after cystectomy and urinary diversion. Int Urol Nephrol.

[7] Launay-Vacher V, Chatelut E, Lichtman SM (2007). Renal insufficiency in elderly cancer patients: International Society of Geriatric Oncology clinical practice recommendations. Ann Oncol.

[8] Baxmann AC, Ahmed MS, Marques NC (2008). Influence of muscle mass and physical activity on serum and urinary creatinine and serum cystatin C. Clin J Am Soc Nephrol.

[9] Raj GV, Iasonos A, Herr H (2006). Formulas calculating creatinine clearance are inadequate for determining eligibility for cisplatin-based chemotherapy in bladder cancer. J Clin Oncol.

[10] Funakoshi Y, Fujiwara Y, Kiyota N (2013). Prediction of glomerular filtration rate in cancer patients by an equation for Japanese estimated glomerular filtration rate. Jpn J Clin Oncol.

[11] Mussap M, Vestra MD, Fioretto P (2002). Cystatin C is a more sensitive marker than creatinine for the estimation of GFR in type 2 diabetic patients. Kidney Int.

[12] Qutb A, Syed G, Tamim HM (2009). Cystatin C-based formula is superior to MDRD, Cockcroft-Gault and Nankivell formulae in estimating the glomerular filtration rate in renal allografts. Exp Clin Transplant.

[13] Knight EL, Verhave JC, Spiegelman D (2004). Factors influencing serum cystatin C levels other than renal function and the impact on renal function measurement. Kidney Int.

[14] Dharnidharka VR, Kwon C, Stevens G (2002). Serum cystatin C is superior to serum creatinine as a marker of kidney function: A meta-analysis. Am J Kidney Dis.

[15] Demirtaş S, Uzunoğlu N, CAN M (2007). Serum cystatin C levels in gastric cancer patients: Scientific letter. Turkiye Klinikleri J Med Sci.

[16] Böike E, Schieren G, Gripp S (2011). Cystatin C - A fast and reliable biomarker for glomerular filtration rate in head and neck cancer patients. Strahlenther Onkol.

[17] Nakai K, Kikuchi M, Fujimoto K (2008). Serum levels of cystatin C in patients with malignancy. Clin Exp Nephrol.

[18] Kos J, Werle B, Lah T (2000). Cysteine proteinases and their inhibitors in extracellular fluids: Markers for diagnosis and prognosis in cancer. Int J Biol Markers.

[19] Andrew S. Levey MDRD GFR Equation - MDCalc.

[20] Journal O, Society I (2013). KDIGO 2012 Clinical Practice Guideline for the Evaluation and Management of Chronic Kidney Disease.

[21] Sjöström P, Tidman M, Jones I (2005). Determination of the production rate and non-renal clearance of cystatin C and estimation of the glomerular filtration rate from the serum concentration of cystatin C in humans. Scand J Clin Lab Invest.

[22] Massey D (2004). Commentary: clinical diagnostic use of cystatin C. J Clin Lab Anal.

[23] Chaudhary SS, Shah JP, Mahato RV (2015). Interference of Bilirubin in Creatinine Value Measurement by Jaffe Kinetic Method. ACCLM.

[24] Nigam PK (2016). Bilirubin Interference in Serum Creatinine Estimation by Jaffe's kinetic Method and Its Rectification in Three Different Kits. Indian J Clin Biochem.

[25] Trimarchi H, Muryan A, Martino D (2012). Creatinine- vs. cystatin C-based equations compared with 99mTcDTPA scintigraphy to assess glomerular filtration rate in chronic kidney disease. J Nephrol.

[26] Giglio D (2014). A new equation for estimating glomerular filtration rate in cancer patients. Chemotherapy.

[27] Benöhr P, Grenz A, Hartmann JT (2006). Cystatin C - A marker for assessment of the glomerular filtration rate in patients with cisplatin chemotherapy. Kidney Blood Press Res.

[28] Feldman HI, Inker LA, Coresh J (2012). Estimating Glomerular Filtration Rate from Serum Creatinine and Cystatin C. N Engl J Med.

[29] Mussap M, Vestra MD, Fioretto P (2002). Cystatin C is a more sensitive marker than creatinine for the estimation of GFR in type 2 diabetic patients. Kidney Int.

[30] LeBricon T, Leblanc I, Benlakehal M (2005). Evaluation of renal function in intensive care: plasma cystatin C vs creatinine and derived glomerular filtration rate estimates. Clin Chem Lab Med.

[31] Ben¨ohr P, Grenz A, Hartmann J (CystatinC-amarker for assessment of the glomerular filtration rate in patients with cisplatin chemotherapy. Kidney Blood Press Res. 2006). T, M¨uller G. A, Blaschke S.

[32] ˇStabuc B, Vrhovec L, ˇStabuc-ˇSilih M (2000). Improved prediction of decreased creatinine clearance byserum cystatin C: use in cancer patients before and during chemotherapy. Clin Chem.

[33] Al-Tonbary YA, Hammad AM, Zaghloul HM (2004). Pretreatment cystatin C in children with malignancy: can it predict chemotherapy-induced glomerular filtration rate reduction during the induction phase?. J Pediatr Hematol Oncol.

[34] Bodnar L, Wcislo GB, Smoter M (2010). Cystatin C as a parameter of glomerular filtration rate in patients with ovarian cancer. Kidney Blood Press Res.

[35] Kume M, Yasui H, Yoshikawa Y (2012). Transient elevation of serum cystatin C concentrations during perioperative cisplatin-based chemotherapy in esophageal cancer patients. Cancer Chemother Pharmacol.

[36] Sutherland GA, Glass J, Gabriel R (1977). Increased incidence of malignancy in chronic renal failure. Nephron.

[37] Launay-Vacher V, Izzedine H, Rey J-B (2004). Incidence of renal insufficiency in cancer patients and evaluation of information available on the use of anticancer drugs in renally impaired patients. Med Sci Monit.

[38] Kutluk Cenik B, Sun H, Gerber DE (2013). Impact of renal function on treatment options and outcomes in advanced non-small cell lung cancer. Lung Cancer.

[39] Tsao CK, Moshier E, Seng SM (2012). Impact of the CKD-EPI equation for estimating renal function on eligibility for cisplatin-based chemotherapy in patients with urothelial cancer. Clin Genitourin Cancer.

[40] Barraclough LH, Field C, Wieringa G (2008). Estimation of renal function - What is appropriate in cancer patients?. Clin Oncol (R Coll Radiol).

[41] Amirrasooli H, Tabatabaeefar M, Moeani B (2015). The efficacy of serum cystatin c and creatinine to diagnose impaired renal function in cancer patients under treatment with cisplatin. UHOD - Uluslararasi Hematol Derg.

